# Supplementary material for: Lidocaine Modulates Cytokine Production and Reprograms the Tumor Immune Microenvironment to Enhance Anti-Tumor Immune Responses in Gastric Cancer
Source: Int J Mol Sci. 2025 Mar 31;26(7):3236. doi: 10.3390/ijms26073236 (PMC11989700; doi:10.3390/ijms26073236)
Supplement: Supplementary file 1 [file ijms-26-03236-s001.zip › ijms-3516683-supplementary.pdf]

Supplementary Materials

Lidocaine Modulates Cytokine Production and Reprograms the Tumor Immune Microenvironment to Enhance Anti-Tumor Immune Responses in Gastric Cancer

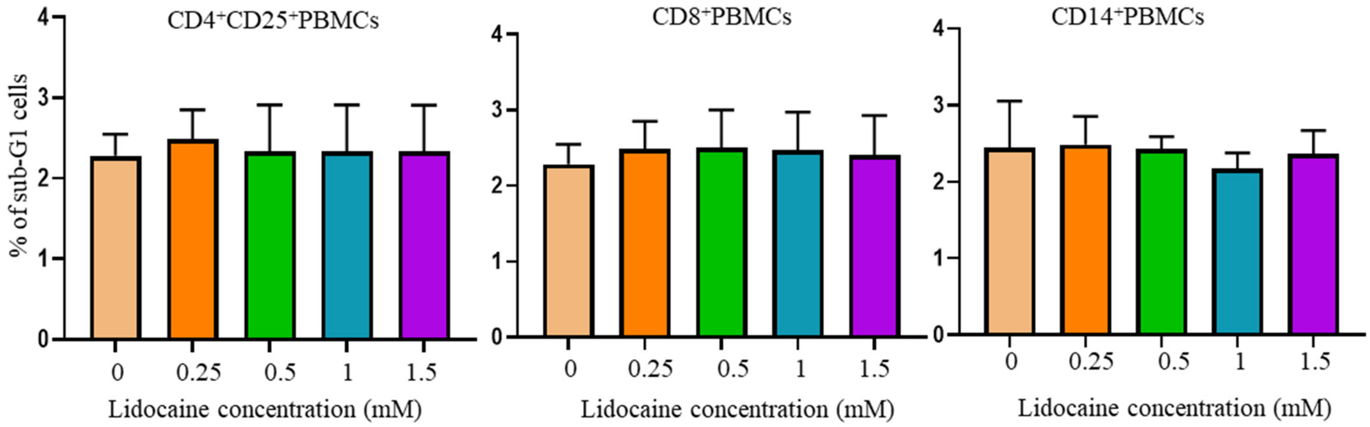

Figure S1. Lidocaine does not affect $CD4^+CD25^+$PBMCs, $CD8^+$PBMCs and $CD14^+$ PBMCs. Human normal $CD4^+CD25^+$PBMCs, $CD8^+$PBMCs and $CD14^+$ PBMCs were assessed by flow cytometric analysis using propidium iodide-stained cells. Firstly, 10^4 cells were incubated in 96-well plates in presence or absence of the indicated concentrations of lidocaine (0.25–1.5 mM). After 72 h treatment, cells were washed with PBS and fixed with 70% ethanol for 1 h on ice. Pelleted cells were incubated with RNase-A (0.1 mg/mL) and propidium iodide (40 μg/mL) for 1 h with shaking and protected from light. The percentage of subG1 population was determined by flow cytometry. Data are representative of three independent experiments; $n \geq 3$.

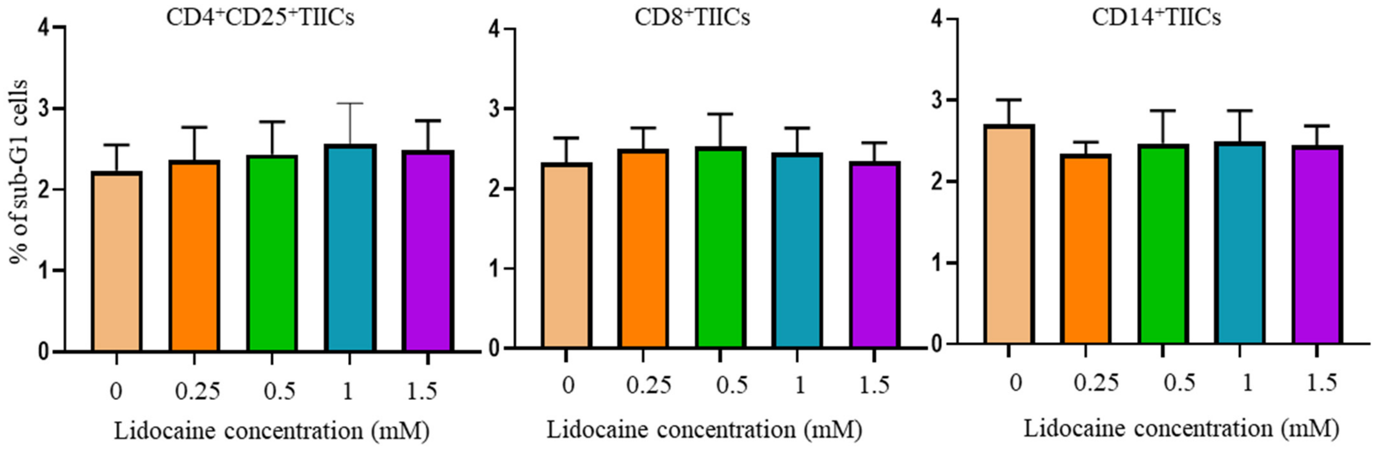

Figure S2. Lidocaine does not affect $CD4^+CD25^+$TIICs, $CD8^+$TIICs and $CD14^+$TIICs viability. Human normal $CD4^+CD25^+$TIICs, $CD8^+$TIICs and $CD14^+$TIICs were assessed by flow cytometric analysis using propidium iodide-stained cells. Firstly, 10^4 cells were incubated in 96-well plates in presence or absence of the indicated concentrations of lidocaine (0.25–1.5 mM). After 72 h treatment, cells were washed with PBS and fixed with 70% ethanol for 1 h on ice. Pelleted cells were incubated with RNase-A (0.1 mg/mL) and propidium iodide (40 μg/mL) for 1 h with shaking and protected from light. The percentage of subG1 population was determined by flow cytometry. Data are representative of three independent experiments; $n \geq 3$.

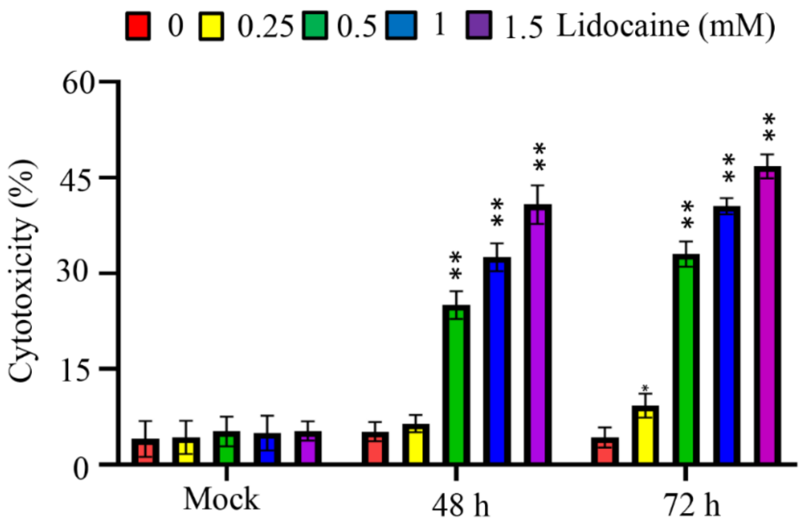

Figure S3. Lidocaine-sensitized CD8⁺TIICs dependent immunogenic cell death of PGCCs. CD8⁺TIICs were co-cultured with PGGCs followed treatment with lidocaine for 48 and 72 h. The ratio of cell numbers of CD8⁺TIICs and PGGCs was 5:1. Cell death was measured using a LDH cytotoxicity assay. Data represent the mean of triplicate experiments, and experiments were repeated at least three times using different donor with similar results. Data are from distinct samples and presented as the mean ± SEM. *$p < 0.05$, **$p < 0.01$ compared with mock treated cells; $n \geq 3$.
